# Laparoscopic enucleation of Frantz's tumor of the pancreas: Case report and literature review

**DOI:** 10.1016/j.amsu.2021.102221

**Published:** 2021-03-14

**Authors:** Dragan Eric, Vladimir Milosavljevic, Mauricio Gonzalez-Urquijo, Boris Tadic, Milan Veselinovic, Nikola Grubor, Djordje Jelic, Miloš Bjelovic

**Affiliations:** a Health Care Polyclinic, Belgrade, Serbia; b Gracia Medica Polyclinic, Belgrade, Serbia; c Tecnologico de Monterrey, School of Medicine and Health Sciences, Ignacio Morones Prieto O 3000, Monterrey, 64710, Mexico; d Clinical Center of Serbia, University Hospital for Digestive Surgery, Department for Minimally Invasive Upper Digestive Surgery, Belgrade, Serbia; e University of Kragujevac, Faculty of Medicine, Kragujevac, Serbia

**Keywords:** Laparoscopic enucleation, Tumor, Pancreas

## Abstract

**Introduction:**

Frantz's tumor of the pancreas is a rare phenomenon, and it accounts for 1–3% of all neoplasms of the pancreas. Its percentage is much higher in younger persons, especially in younger women, as compared to the rest of the population.

**Presentation of case:**

The present study describes a 32-year-old female patient in whom a preoperative imaging diagnosis confirmed a mass in the junction of the pancreas' body and tail. Based on the anamnesis, the preoperative diagnosis, and the patient's general status, the decision was made to performed laparoscopic enucleation of the pancreatic tumor. The operation and postoperative recovery passed without complications. Definitive histopathological and immunohistochemical findings confirmed a solid pseudopapillary neoplasm of the pancreas.

**Discussion:**

Depending on the localization and the size of the tumor, surgical options range from typical and atypical resections of the pancreas to minimally invasive surgical procedures, such as local excision and enucleation. Laparoscopic procedures have a comparative advantage in cases of enucleation and resection of the pancreas. The low frequency of recidivation and a favorable prognosis, even after repeated surgery, are additional reasons for favoring the laparoscopic approach over the classical surgical approach.

**Conclusion:**

A minimally invasive surgical approach should be applied whenever the dimensions and the localization of the tumor permit it, bearing in mind all the benefits and advantages that this surgical technique offers.

## Highlights

• Solid pseudopapillary neoplasm of the pancreas, is known as Frantz's tumor.
• Around 1,000 cases of Frantz's tumor have been described.
• 32-year-old patient underwent laparoscopic enucleation of an 18 mm pancreatic tumor.
• Laparoscopic enucleation is a feasible option for the treatment of these masses.

## 1 Introduction

Solid pseudopapillary neoplasm of the pancreas, also known as Frantz's tumor, is a rare phenomenon, and it accounts for 1–3% of all neoplasms of the exocrine pancreas. It mainly occurs in younger individuals, predominantly women [1]. Non-specific symptomatology is often present, such as abdominal pain or sporadic manifestations of nausea, vomiting, and a sense of heaviness in the abdomen. In rare cases, at physical examination, a palpable mass may be present in the abdomen [2]. Frantz's tumor may be localized in any part of the pancreas. There are rarely metastases, and when they do occur, they are most commonly localized in the liver [1,2].

Ultrasonography (USG) of the abdomen is the initial diagnostic method; nevertheless, computerized tomography (CT) and magnetic resonance imaging (MRI) of the abdomen are much more precise and superior methods. Frequently, CT and MRI can indicate the presence of a mass in the pancreas, which has the characteristics of cystic degeneration, bleeding within the cyst, and the presence of a capsule [3]. Differential diagnoses include pancreatic ductal adenocarcinoma, cystadenoma, cystadenocarcinoma, or a pancreatic neuroendocrine tumor [4].

The only curative treatment modality is surgical treatment. Within this modality, the available options range from resection procedures on the pancreas, depending on the tumor's localization, to a minimally invasive surgical procedure on the pancreas in the form of the enucleation of these neoplasms [5].

The present study aims to present a rare disease of the pancreas, which was treated safely and efficiently by applying laparoscopic enucleation with all the benefits this approach offers when used appropriately. A review of the literature is also performed. The work has been reported in line with the SCARE guidelines [6].

## 2 Presentation of case

**Fig. 1:**
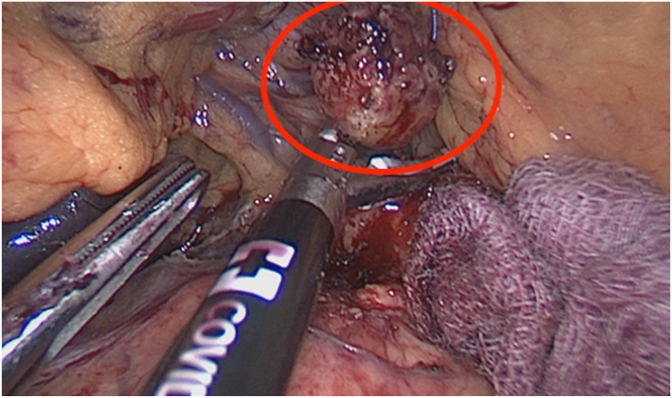
MRI showing a 18 mm pancreatic mass, in the junction of the corpus and tail of the pancreas. Red arrow pointing towards the tumor. (For interpretation of the references to colour in this figure legend, the reader is referred to the Web version of this article.)

A 32-year-old patient with no past medical history and no comorbidities presented to the emergency room, referring to abdominal pain below the left costal arch. On physical examination, the patient was hemodynamically stable and afebrile. After a thorough abdominal exploration, no positive signs for peritonism were detected. Laboratories showed a hemoglobin of 13.7 g/dl, a normal white blood count of 5.4 × 10^9^. No other alterations were encountered. An abdominal ultrasound was done, without any abnormal findings, so an MRI was ordered, detecting a focal area with a distortion in the parenchyma, diagnosing an 18 mm pancreatic mass in the junction of the corpus and tail of the pancreas. There were no signs of infiltration of the surrounding vascular structures and no connection to the duct of Wirsung (Fig. 1). As to differential diagnosis, the lesion had the characteristics of a pancreatic neuroendocrine tumor, a pseudotumor, or an intrapancreatic splenunculum. Tumor markers were within the reference ranges. After tumor board consultation, the decision to operated on the patient was carried out.

**Fig. 2:**
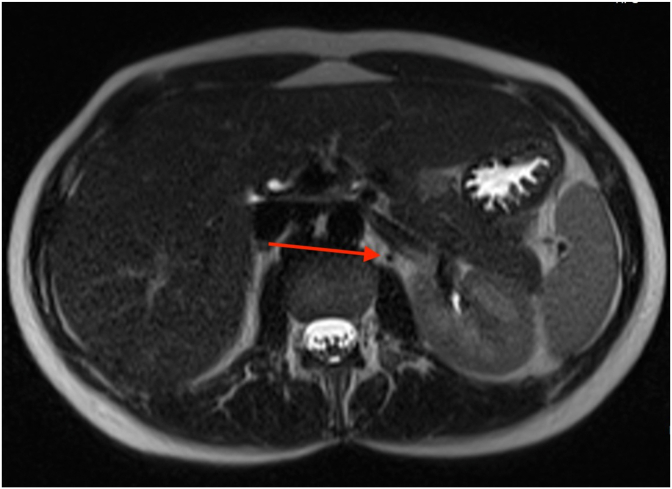
Enucleation of the pancreatic mass. The red circle shows the tumor. (For interpretation of the references to colour in this figure legend, the reader is referred to the Web version of this article.)

The patient was then transferred to the operating room, where she was put under general endotracheal anesthesia. The attending on-call performed an artificial pneumoperitoneum, and after working ports were placed on the sites, optical instruments were inserted, and abdominal exploration was made. The exploration verified a normal finding of the abdomen without signs of dissemination of the disease on the liver or the serous membranes and without any free fluid in the abdomen. The omental bursa was opened via the intercoloepiploic access, and the anterior aspect of the pancreas body was explored. A mass in the junction of the body and the tail of the pancreas of 20 mm in diameter was encountered, and an enlarged lymph gland around the coronary vein, which was completely removed and sent for extempore histopathological analysis, with a benign finding. Afterward, enucleation of the entire pancreatic mass was performed using the LigaSure device (SurgRx, Redwood City, CA) (Fig. 2). A drain was placed into the omental bursa, the gas was let out, and the surgical incisions were reconstructed by anatomical layers. The sample was sent for final histopathological diagnosis.

Postoperative recovery went uneventful. The abdominal drain was removed on the third postoperative day. On the fourth postoperative day, the patient was discharged from the hospital with normal laboratory findings. A month after surgery, ultrasound imaging of the patient's abdomen was performed, and without abnormalities. At this time, laboratory analyses were also performed, and the results were within the normal reference ranges. Six months after the operation, a follow-up MRI of the abdomen was conducted without any abnormal findings. The patient is currently on her 12 month follow-up without complications nor recurrence.

**Fig. 3:**
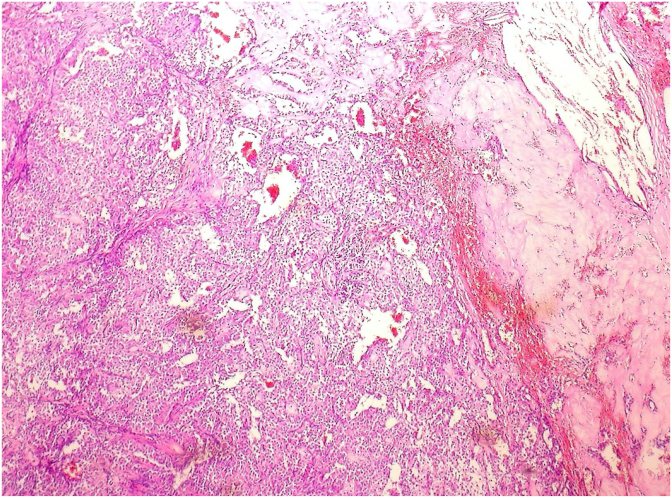
Histological sections of the tumor show the characteristic organization of the tumor with vascular pseudopapillae and loose arrangement of weakly cohesive cells, irregular microcysts, and cholesterol crystals surrounded by fibrotic stroma and histiocytes.

Definitive histopathological and immunohistochemical findings confirmed a solid pseudopapillary neoplasm of the pancreas. The tumor did not show cytological or histological elements of malignancy (Fig. 3).

## 3 Discussion

The solid pseudopapillary neoplasm of the pancreas was first mentioned in the literature in 1933 [5]. However, as a separate phenomenon, with low malignant potential, it was introduced by Virginia Kneeland and Frantz in 1959 [7]. The prevalence of these neoplasms is higher in children and younger individuals, aged 18–35 years. It is more frequent in the female sex, with a ratio of 10:1, compared to the male sex. In several cases described in the literature, male patients aged over 60 were diagnosed with this tumor [2]. A review of the literature shows that, so far, around 1,000 cases of Frantz's tumor have been described [2,8].

These tumors most commonly remain asymptomatic or cause non-specific complaints, regardless of their size. In addition to the compressive effect on the surrounding structures and the occurrence of a palpable mass in the abdomen once they reach a specific size, occasional acute or chronic pain, loss of appetite, vomiting, and a loss in body mass, may also occur [9].

In the patient described in the present study, symptoms were present around two months, in non-specific pain below the left costal arch. The pain was of lower intensity, more discomfort in that part of the abdomen, and occasional intensification in the pain scale.

Appropriate diagnosis plays an essential role in deciding on further treatment. The initial diagnostic methods applied are US and CT of the abdomen. A heterogeneous lesion can be seen in the latter, which often demonstrate a peripheral enhancement of contrast, characteristic of the fibrous pseudocapsule. MRI of the abdomen is a more sensitive method than CT [8]. Some authors attribute great importance to endoscopic ultrasound (EUS), which can provide more precise localization and more accurate insight into the relations between the mass and the surrounding organ and vascular structures. EUS has the advantage of performing a fine-needle biopsy, with a low risk of possible complications, and can provide more precise diagnostic data than the aforementioned imaging procedures [10].

Frantz's tumor has a low malignant potential. There are rarely metastases, and they are most commonly localized in the liver. Even when there are metastases, the overall 5-year survival is around 97% [11].

Surgery is the only and primary curative form of treatment. Depending on the localization and the size of the tumor, surgical options range from typical and atypical resections of the pancreas to minimally invasive surgical procedures, such as local excision and enucleation [12]. Laparoscopic procedures have a comparative advantage in cases of enucleation and resection of the pancreas. The low frequency of recidivation and a favorable prognosis, even after repeated surgery, are additional reasons for favoring the laparoscopic approach over the classical surgical approach [2,13].

Due to the favorable localization of the tumor, its size, and bearing in mind all the advantages of a minimally invasive surgical approach, we chose a laparoscopic enucleation of the tumor, which went uneventfully.

## 4 Conclusion

With its mainly non-specific symptomatology, Frantz's tumor represents a diagnostic challenge and is usually detected as a coincidental finding during other examination procedures. As it mostly occurs in the younger population, especially in younger women between 18 and 35 years old, one must consider the best therapeutic approach to treat this tumor. Laparoscopic enucleation is a feasible option for the treatment of these masses.

## Consent

Written informed consent was obtained from the patient for publication of this case report and accompanying images. A copy of the written consent is available for review by the Editor-in-Chief of this journal on request.

## Ethical approval

All procedures performed in this study were reviewed by Ethics Committee and complied with the principles laid down in the Declaration of Helsinki.

## Funding

This research group did not receive any specific grant from funding agencies in the public, commercial, or not-for-profit sectors.

## Guarantor

Dr. Mauricio Gonzalez-Urquijo.

## Author contribution

Conceptualization, D.E.; V.M; M.G.U; Writing-review and editing, B.T, M.V; N.G; D.J Literature review, M.B.; V.M; M.G.U; B.T Writing draft, M.V.; M.G.U, D.E.; Supervision, M.G.U.

## Provenance and peer review

Not commissioned, externally peer-reviewed.

## Ethical approval

Ethics Committee of the Clinical Center of Tecnologico de Monterrey.

DECISION: We approve the academic clinical work **Laparoscopic Enucleation of Frantz's Tumor of the Pancreas: Case Report and Literature Review** to be conducted.

No. 01134TM.

Date: 26/01/2021.

## Registration of research studies

Name of the registry: Laparoscopic Enucleation of Frantz's Tumor of the Pancreas.

Unique Identifying number or registration ID: NCT04728750.

Hyperlink to your specific registration (must be publicly accessible and will be checked): https://clinicaltrials.gov/show/NCT04728750.

## Declaration of competing interest

None.
